# Supplementary material for: Modeling glioblastoma heterogeneity as a dynamic network of cell states
Source: Mol Syst Biol. 2021 Sep 16;17(9):e10105. doi: 10.15252/msb.202010105 (PMC8444284; doi:10.15252/msb.202010105)
Supplement: Supplementary file 6 — Source Data for Figure 5 [file MSB-17-e10105-s004.zip › Figure5A_sourcedata/GSEA_3017/nosigdb_stateD.GseaPreranked.1622795010397/PARADA_T1_UP_26461091.html]

Details for gene set PARADA\_T1\_UP\_26461091[GSEA]

|  || Dataset | state23017 |
| Phenotype | NoPhenotypeAvailable |
| Upregulated in class | na\_neg |
| GeneSet | PARADA\_T1\_UP\_26461091 |
| Enrichment Score (ES) | -0.61174047 |
| Normalized Enrichment Score (NES) | -2.6085815 |
| Nominal p-value | 0.0 |
| FDR q-value | 0.0 |
| FWER p-Value | 0.0 |
Table: GSEA Results Summary

  

Fig 1: Enrichment plot: PARADA\_T1\_UP\_26461091      
 Profile of the Running ES Score & Positions of GeneSet Members on the Rank Ordered List

  

| PROBE | GENE SYMBOL | GENE\_TITLE | RANK IN GENE LIST | RANK METRIC SCORE | RUNNING ES | CORE ENRICHMENT || 1 | VIM |  |  | 75 | 0.314 | -0.0381 | No |
| 2 | TROAP |  |  | 109 | 0.283 | -0.0456 | No |
| 3 | BIRC5 |  |  | 130 | 0.271 | -0.0439 | No |
| 4 | MCM2 |  |  | 226 | -0.256 | -0.1011 | No |
| 5 | PARP14 |  |  | 339 | -0.267 | -0.1708 | No |
| 6 | RNF213 |  |  | 533 | -0.289 | -0.3018 | No |
| 7 | PRC1 |  |  | 645 | -0.310 | -0.3680 | No |
| 8 | PSMB8 |  |  | 739 | -0.328 | -0.4190 | No |
| 9 | ANXA2 |  |  | 777 | -0.338 | -0.4262 | No |
| 10 | HIST1H2AG |  |  | 786 | -0.339 | -0.4108 | No |
| 11 | CKAP2 |  |  | 900 | -0.375 | -0.4744 | No |
| 12 | RHOJ |  |  | 904 | -0.376 | -0.4528 | No |
| 13 | LHFPL3 |  |  | 919 | -0.381 | -0.4393 | No |
| 14 | BUB1B |  |  | 1111 | -0.477 | -0.5568 | No |
| 15 | KIF2C |  |  | 1183 | -0.558 | -0.5762 | Yes |
| 16 | CDCA3 |  |  | 1187 | -0.563 | -0.5427 | Yes |
| 17 | GTSE1 |  |  | 1210 | -0.602 | -0.5214 | Yes |
| 18 | NCAPH |  |  | 1228 | -0.643 | -0.4936 | Yes |
| 19 | CENPA |  |  | 1243 | -0.679 | -0.4612 | Yes |
| 20 | TAGLN2 |  |  | 1247 | -0.698 | -0.4191 | Yes |
| 21 | CDCA2 |  |  | 1258 | -0.727 | -0.3806 | Yes |
| 22 | HMGB2 |  |  | 1263 | -0.736 | -0.3369 | Yes |
| 23 | KIF23 |  |  | 1265 | -0.741 | -0.2905 | Yes |
| 24 | AURKA |  |  | 1271 | -0.763 | -0.2458 | Yes |
| 25 | SPP1 |  |  | 1277 | -0.787 | -0.1996 | Yes |
| 26 | IFITM3 |  |  | 1279 | -0.812 | -0.1487 | Yes |
| 27 | LGALS3 |  |  | 1287 | -0.878 | -0.0982 | Yes |
| 28 | HIST2H2AC |  |  | 1288 | -0.880 | -0.0422 | Yes |
| 29 | HIST1H2AH |  |  | 1305 | -1.040 | 0.0116 | Yes |
Table: GSEA details [plain text format]

  

Fig 2: PARADA\_T1\_UP\_26461091: Random ES distribution      
 Gene set null distribution of ES for **PARADA\_T1\_UP\_26461091**
